# Association of Helicobacter pylori in Children With Self-Hand Hygiene, Maternal Hand Hygiene, Cooking, and Feeding Practices

**DOI:** 10.7759/cureus.56554

**Published:** 2024-03-20

**Authors:** Janhavi V Thorat, Sampada Tambolkar, Mukta M Chitale, Vishnu Biradar, Renuka S Jadhav

**Affiliations:** 1 Pediatrics, Dr. D. Y. Patil Medical College, Hospital and Research Centre, Dr. D. Y. Patil Vidyapeeth, Pune, IND; 2 Pediatric Gastroenterology, Dr. D. Y. Patil Medical College, Hospital and Research Centre, Dr. D. Y. Patil Vidyapeeth, Pune, IND

**Keywords:** maternal hygiene, nail hygiene, hand hygiene, food hygiene, helicobacter pylori

## Abstract

Background and objective

*Helicobacter pylori* infection is widely prevalent, but its route of transmission is not clear. Person-to-person transmission seems plausible, with hand hygiene being one of the many factors that play a role.

The objective of this study was to study the effect of the children’s and their mother’s hand hygiene and feeding practices on the prevalence of *H. pylori* in children.

Methodology

This cross-sectional study involved 475 children and their mothers. A questionnaire was administered to mothers to gather information about maternal hygiene practices, specifically handwashing before food handling and after using the toilet. Additionally, both mothers and children underwent assessments for nail length (whether cut or uncut) and the presence or absence of dirt under their nails, if nails were uncut. The association of these parameters with *H. pylori* seropositivity in children was comprehensively examined. Furthermore, children were divided into two distinct groups: a younger age group (one month to two years and 11 months) and an older age group (three years to 15 years). For one specific parameter - the presence of dirt under mothers' nails (i.e., if nails were uncut) - the association was further analyzed separately within these age groups. The chi-square test was applied to all variables. *P *< 0.05 was considered significant

Results

The association of all variables with *H. pylori* seropositivity in children was tested. Association with *H. pylori* seropositivity was not present in mothers with uncut nails (*P* = 0.050315), mothers with uncut nails harboring dirt under their nails within the entire sample of 475 mothers (*P* = 0.39476), and mothers with uncut nails harboring dirt under their nails in the older age group (three years to 15 years) of children (*P* = 0.760071). Association with *H. pylori* seropositivity was present in mothers with dirt under their uncut nails belonging to the younger age group of children (one month to two years and 11 months (*P* = 0.014127) and mothers who did not wash their hands before food handling (*P *= 0.003032) and after using the toilet (*P* = 0.003082). In all 475 children, association with *H. pylori* seropositivity was significant with dirt under the uncut nails of children (*P *= 0.015194) and was not significant for children with merely grown nails but not harboring dirt under them (*P *= 0.355967).

Conclusions

Mother-to-child transmission is one of the likely routes of transmission of *H. pylori*, and poor hand hygiene seems to play a major role in this process.

## Introduction

*Helicobacter pylori *is a microaerophilic flagellated bacterium that does not take up the Gram stain. It was identified in 1984 [1]. Its prevalence remains high in developing countries [2]. A comparatively older study by Gill et al. suggests that around 83% of the Indian pediatric population is exposed to *H. pylori* in the first few years of life [3]. The infection is acquired in childhood and, once acquired, stays for life. Human saliva, dental plaque, and feces are known to harbor *H. pylori *[4]. Transmission from infected mothers has been implicated in some other studies [5,6]. The prevalence of infection is higher in sexual partners of individuals infected with *H. pylori*, highlighting the importance of close contact and cohabitation [7]. The institutional and familial clustering of *H. pylori* infection further underlines the role of nongenetic and environmental factors in the infective process [8,9].

Tertiary or higher levels of education are known to be protective factors against *H. pylori* positivity [3,10]. Lower socioeconomic status, poor living conditions, and the absence of breastfeeding have emerged as independent risk factors for infection in a study conducted on the Turkish pediatric population [11]. However, these remain controversial, as some studies find no such associations [12]. Infrequent eating with hands is associated with a decreased risk of infection, which points to hand hygiene as an important risk factor [13]. 

This study aims to establish a relationship between the hand hygiene of children, their mothers, and the prevalence of *H. pylori* infection in the pediatric population.

## Materials and methods

Study design

There are two types of *H. pylori*, gastric and enterohepatic. This study pertains to gastric *H. pylori* [1]. This is a cross-sectional study conducted at a tertiary-level hospital in Pune, India, which included children between one month and 15 years old. Written informed consent was obtained from the participants’ guardians. 

The study conducted by Aitila et al. showed the prevalence of *H. pylori* infection among study participants to be 24.3% [14]. Entering these data into the Win-Pepi software and taking an allowable error of 5% at a 95% confidence interval, the calculated sample size came to 283. Considering the nonresponse rate to be 10%, the total sample size was 315. However, we studied 475 subjects and their mothers.

The children were residents of Western Maharashtra and were visiting the general pediatrics outpatient department of the hospital for various reasons, including but not limited to immunization, illnesses, and procedures.

Inclusion criteria

All children between one month and 15 years whose guardians were willing to participate in the study were included.

Exclusion criteria

All children who had already been tested for *H. pylori* and treated, if positive, were excluded.

*Helicobacter pylori* detection method

The Onsite H. pylori Antibody Combo Rapid Test is a sandwich lateral flow chromatographic immunoassay that qualitatively detects antibodies (IgG, IgM, and IgA) against *H. pylori* in human plasma, serum, or whole blood. This is a card test with a test-to-result time of 15 minutes, with a visual read. One single sample of 1 mL venous blood was analyzed for IgG, IgM, and IgA through the aforementioned test.

Data collection

Data were collected from the outpatient department (OPD) of a tertiary care hospital. The data were primarily divided into two groups: children with a positive *H. pylori*-rapid assay test and children with a negative *H. pylori*-rapid assay test. Data were obtained on whether the subjects' mothers washed their hands before meals, after using the toilet, and after contamination due to any reason through a questionnaire.

The children were then divided into two groups based on age: the first group consisted of children from age one month to two years and 11 months (younger age group), and the second group consisted of children from ages three years to 15 years (older age group).

Mothers of children from each group were assessed to determine whether they had grown or cut nails. Among them, 257 (54%) mothers had grown/uncut nails, while 218 (46%) mothers had cut nails. The association between cut/uncut nails of mothers and *H. pylori* seropositivity was examined. The presence of dirt under the nails of the 257 (54%) mothers with grown/uncut nails was examined. The association between the subset of mothers who had not cut their nails (257/475, 54%) and harbored dirt under their uncut nails (123/257, 48%mothers) and *H. pylori* seropositivity was analyzed across the study population with uncut nails (257/475, 54% mothers). Further stratified analysis was conducted by dividing the study population into two age groups. In the first age group, comprising 108 mothers (42% of the total 257 mothers), 52 (48%) had dirt under their uncut nails. In the second age group, consisting of 149 mothers (58% of the total 257 mothers), 71 (48%) had dirt under their uncut nails, as mentioned earlier.

The association of *H. pylori* positivity with cut nails (288/475, 61% children) or uncut nails (187/475, 39% children) of children as a whole (all 475 participants) was also assessed. In children with uncut nails (187/475, 39% children), an association between dirt under nails (105/187, 56% children) and *H. pylori* seropositivity was noted.

The association of *H. pylori* seropositivity in children (both age groups combined) with mothers washing or not washing their hands before handling food and after the use of the toilet or any contamination was assessed.

Data analysis

The chi-square test was applied to the groups. The *P*-value <0.05 was considered significant, according to IBM SPSS Statistics for Windows, Version 28.0 (IBM Corp., Armonk, NY).

## Results

There was no significant association between mothers with grown nails and *H. pylori-*positive children (Table 1).

**Table 1:** Association between mothers with grown nails and H. pylori seropositivity in children.

	*Helicobacter pylori*-positive children, *n* (%)	*H. pylori-*negative children, *n* (%)	Marginal rows total, *n* (%)	*P*-value
Mothers whose nails were not cut	86 (18%)	171 (36%)	257 (54%)	0.05
Mothers whose nails were cut	55 (12%)	163 (34%)	218 (46%)	
Marginal column totals	141 (30%)	334 (70%)	475 (100%)	

Only those mothers who had uncut nails, including 257 out of the total 475 (54%) mothers, were assessed for dirt under their nails. In the overall population, without division into two age groups as mentioned in the Methods section, no statistically significant association was found between dirt under the nails of the mothers and *H. pylori* seropositivity (Table 2).

**Table 2:** Association between maternal nail hygiene and H. pylori seropositivity in patients.

	*Helicobacter pylori*-positive children, *n* (%)	*H. pylori*-negative children, *n* (%)	Marginal row totals, *n* (%)	*P*-value
Dirt present under the nails of the mothers with uncut nails	45 (18%)	78 (30%)	123 (48%)	0.39
No dirt present under the nails of the mothers with uncut nails	41 (16%)	93 (36%)	134 (52%)	
Marginal column totals	86 (34%)	171 (66%)	257 (100%)	

Out of the total children with mothers having uncut nails (257), 108 (42%) were from the younger age group, comprising children aged one month to two years and 11 months. These mothers were examined for dirt under their uncut nails. There was a statistically significant association of dirt under the nails of mothers with *H. pylori *seropositivity in the children (Table 3).

**Table 3:** Association between maternal nail hygiene and H. pylori seropositivity in younger patients. Age of patients: one month to two years and 11 months.

	*Helicobacter pylori*-positive children, *n* (%)	*H. pylori*-negative children, *n* (%)	Marginal row totals, *n* (%)	*P*-value
Dirt present under the nails of the mothers with uncut nails	14 (13%)	38 (35%)	52 (48%)	0.014127
No dirt present under the nails of the mothers with uncut nails	5 (5%)	51 (47%)	56 (52%)	
Marginal column totals	19 (18%)	89 (82%)	108 (100%)	

Out of the total children with mothers having uncut nails (257), 149 (58%) were from the older age group, namely, children aged three to 15 years. These mothers were examined for dirt under their uncut nails. There was no statistically significant association between dirt under the nails of mothers and *H. pylori* seropositivity in the children (Table 4).

**Table 4:** Association between maternal nail hygiene and H. pylori seropositivity in older children. Age of patients: three to 15 years.

	*Helicobacter pylori*-positive children, *n* (%)	*H. pylori*-negative children, *n* (%)	Marginal row totals, *n* (%)	*P*-value
Dirt present under the nails of the mothers with uncut nails	31 (21%)	40 (27%)	71 (48%)	0.76
No dirt present under the nails of the mothers with uncut nails	36 (24%)	42 (28%)	78 (52%)	
Marginal column totals	67 (45%)	82 (55%)	149 (100%)	

For mothers who did not wash their hands before food handling, there was a statistically significant association with *H. pylori* seropositivity in children of both age groups together (Table 5).

**Table 5:** Association between maternal hand hygiene before food handling and H. pylori seropositivity in the entire patient population.

	*Helicobacter pylori*-positive children, *n* (%)	*H. pylori*-negative children, *n* (%)	Marginal rows total, *n* (%)	*P*-value
Mothers who washed their hands before food handling	55 (12%)	180 (38%)	235 (50%)	0.003032
Mothers who do not wash their hands before food handling	86 (18%)	154 (32%)	240 (50%)	
Marginal column totals	141 (30%)	334 (70%)	475 (100%)	

For mothers who did not wash their hands after using the toilet, there was a statistically significant association with *H. pylori *seropositivity in their children for both age groups combined (Table 6)

**Table 6:** Association between maternal hand hygiene after using the toilet and H. pylori seropositivity in patients.

	*Helicobacter pylori*-positive children, *n* (%)	*H. pylori*-negative children, *n* (%)	Marginal row totals, *n* (%)	*P*-value
Mothers who wash hands after using the toilet/ after contamination	58 (12%)	187 (39%)	245 (52%)	0.003082
Mothers who do not wash their hands after using the toilet/after contamination	83 (17%)	147 (31%)	230 (48%)	
Marginal column totals	141 (30%)	334 (70%)	475 (100%)	

No statistically significant association was found between uncut nails of children and *H. pylori* seropositivity (Table 7).

**Table 7:** Association between nail growth and H. pylori seropositivity in patients.

	*Helicobacter pylori*-positive children, *n* (%)	*H. pylori*-negative children, *n* (%)	Marginal row totals, *n* (%)	*P*-value
Children with nails cut	81 (17%)	207 (43%)	288 (60%)	0.35
Children with grown nails	60 (13%)	127 (27%)	187 (40%)	
Marginal column totals	141 (30%)	334 (70)	475 (100%)	

Out of 475 children, 187 (39%) had uncut nails, of which 105 (22%) children had dirt under their nails. There was a statistically significant association between dirt under the nails of children (both younger and older age groups) and *H. pylori* seropositivity (Table 8).

**Table 8:** Association between nail hygiene and H. pylori seropositivity in patients.

	*Helicobacter pylori*-positive children, *n* (%)	*H. pylori*-negative children, *n* (%)	Marginal row totals, *n* (%)	*P*-value
Dirt present under the uncut nails of children	26 (14%)	79 (42%)	105 (56%)	0.015194
No dirt present under the uncut nails of children	34 (18%)	48 (26%)	82 (44%)	
Marginal column totals	60 (32%)	127 (68%)	187 (100%)	

## Discussion

Our research shows that there is an association between *H. pylori* infection with poor hand hygiene pertaining to both the subject and their caregiver. Multiple studies showed that parental transmission of *H. pylori* is very much possible and prevalent [9,15]. The study by Drumm et al., on North American children, showed a strong association between infection in mothers and *H. pylori* colonization in their children [9]. A study by Liu et al., on the Chinese population, suggested maternal infection as a risk factor for a child to be infected [16]. A study by Weyermann et al. suggested that infected mothers are the main source of *H. pylori* infection for their children [17]. A Japanese follow-up study by Malaty et al. showed that the prevalence of *H. pylori *infection was four times higher among children living with positive index mothers than children living with negative index mothers [6]. Hence, it can be said that maternal infection is a risk factor for transmission of *H. pylori*, which also highlights that close contact may provide a favorable route for *H. pylori* to infect children. Though the route of transmission is unclear, we have established evidence with our study that poor hand hygiene is a link in the infective process. *H. pylori* infection was associated with the presence of dirt under the nails of the children in our study. A similar result was observed in the mothers of the children from the younger age group who had dirt present under their uncut nails. In a study by Dowsett et al., samples collected from below the nail of the index fingers of subjects, 58% tested positive for *H. pylori* polymerase chain reaction (PCR), though the study also showed a weak association of PCR nail positivity and *H. pylori* seropositivity [18]. This finding raises the intriguing possibility that the area under the nails could potentially serve as a reservoir for *H. pylori* and, therefore, remains an open question in research. 

Although the association between the presence of dirt under the mothers' uncut nails and *H. pylori* seropositivity in their children was significant for children of the younger age group, it was not significant for children from three to 15 years (older age group). It may be possible because the younger age group from one month to two years and 11 months is more dependent on their mothers for their daily needs, including regular feeding and personal hygiene. A child from the younger (first) age group is in more proximity to their primary caregiver than the second age group. 

Infrequent handwashing was identified as a risk factor for *H. pylori* infection according to Goto et al. [13]. Our study also demonstrated that mothers not washing their hands before handling food was associated with *H. pylori* infection in children in the younger age group. A study by Chen et al. in Hainan, China, showed that handwashing before meals is a protective factor against infection [19]. Another Chinese study by She et al., on the other hand, found no relation between handwashing before and after meals and *H. pylori* infection [12]. Our study along with the former studies attests to the fact that personal hygiene of the primary caregiver plays a role in the transmission of *H. pylori* infection. On the other hand, the older age group is comparatively more independent of their caregiver in terms of personal care and feeding, in this case, their mother, and hence may have remained unaffected despite their mothers practicing poor hygiene. Che et al. demonstrated, similar to our study, that not washing hands after using the toilet was a risk factor for *H. pylori* infection [3]. There was a statistically significant association between handwashing routinely after the use of the toilet by the mother and *H. pylori* seropositivity in the children in our study. A study by Miranda et al., in which the higher age group had more prevalence, suggested that most children acquire the infection when they are young and dependent, and there is a possibility of breaking this chain of transmission through hand hygiene practices [20]. There is ample evidence, including our study, to say that hand hygiene plays a major role in the transmission of *H. pylori* infection. This points to the importance that must be given to the person-to-person transmission of *H. pylori* infection. Also, a simple practice like handwashing, which can be easily and widely implemented, will concurrently reduce the transmission and incidence of diarrhoeal disease and respiratory infections like the COVID-19 virus and help in the reduction of the total healthcare burden on the governance system.

The use of a rapid card test, instead of more traditional diagnostic approaches such as the urea breath test or endoscopy, is one of the limitations of our study. Furthermore, the participants were primarily drawn from those already present at the hospital for various reasons (such as accompanying their parents for parents’ doctor visits, some visiting for immunization, and others visiting for a variety of symptoms, including gastrointestinal symptoms). This may not accurately represent the broader community, potentially impacting the scope to extrapolate our findings to the large community population. A more comprehensive follow-up study involving community testing can be done to further extend the findings of this study. Another limitation is the potential for acquired passive immunity in children aged under six months, hence rendering the test positive. However, in an attempt to reduce inaccuracy pertaining to this, children less than one month of age were excluded from the study.

## Conclusions

The vulnerability of the younger population, specifically children aged one month up to two years and 11 months, could be possibly explained due to their increased susceptibility to infection through caregiver contact. As a targeted strategy, public education efforts for primary prevention should prioritize this age group, imparting knowledge about the importance of handwashing and maintaining clean nails. These initiatives could potentially disrupt the transmission chain and ensure a healthier future for children. Additionally, the potential for mother-to-child transmission of *H. pylori* emphasizes the significance of addressing the role of maternal hand hygiene in this transmission pathway. The behavioral change of regular handwashing before food handling and after contamination emerges as a simple yet impactful intervention. The often-overlooked reservoir for infection, dirt under the nails, adds a layer of complexity to the transmission dynamics, emphasizing the need for increased awareness and preventive measures.
